# Clinical features of idiopathic inflammatory polymyopathy in the Hungarian Vizsla

**DOI:** 10.1186/s12917-015-0408-7

**Published:** 2015-04-21

**Authors:** Anna Tauro, Diane Addicott, Rob D Foale, Chloe Bowman, Caroline Hahn, Sam Long, Jonathan Massey, Allison C Haley, Susan P Knowler, Michael J Day, Lorna J Kennedy, Clare Rusbridge

**Affiliations:** Fitzpatrick Referrals, Halfway Lane, Eashing, Godalming, GU7 2QQ Surrey UK; Murrayfield, Lockerbie, UK; Dick White Referrals, Six Mile Bottom, Suffolk, UK; Adelaide Veterinary Specialist and Referral Centre (AVSARC), Norwood Adelaide, South Australia; Royal (Dick) School of Veterinary Studies, University of Edinburgh, Roslin, UK; CIGMR, The University of Manchester, Manchester, UK; The University of Georgia, College of Veterinary Medicine, Athens, USA; University of Bristol, Langford, Bristol, UK; The University of Surrey, Guildford, Surrey UK

**Keywords:** Regurgitation, Dysphagia, Canine, Dog Leukocyte Antigen, Familial polymyositis

## Abstract

**Background:**

A retrospective study of the clinicopathological features of presumed and confirmed cases of idiopathic inflammatory polymyopathy in the Hungarian Vizsla dog and guidelines for breeding.

**Results:**

369 medical records were reviewed (1992–2013) and 77 Hungarian Vizslas were identified with a case history consistent with idiopathic inflammatory polymyopathy. Inclusion criteria were: group 1 (confirmed diagnosis); histopathology and clinical findings compatible with an inflammatory polymyopathy and group 2 (probable diagnosis); clinical findings compatible with a polymyopathy including dysphagia, sialorrhea, temporal muscle atrophy, elevated serum creatine kinase (CK) activity, and sufficient clinical history to suggest that other neuromuscular disorders could be ruled out. Some group 2 dogs had muscle biopsy, which suggested muscle disease but did not reveal an inflammatory process. The mean age of onset was 2.4 years; male dogs were slightly overrepresented. Common presenting signs were dysphagia, sialorrhea, masticatory muscle atrophy, and regurgitation. Common muscle histopathological findings included degenerative and regenerative changes, with multifocal mononuclear cell infiltration with lymphoplasmacytic myositis of variable severity. A positive response to immunosuppressive treatment supported an immune-mediated aetiology. The mean age at death and survival time were 6.4 and 3.9 years, respectively. Recurrence of clinical signs and aspiration pneumonia were common reasons for euthanasia.

**Conclusions:**

Diagnosis of Vizsla idiopathic inflammatory polymyopathy can be challenging due to lack of specific tests, however the presence of dysphagia, regurgitation and masticatory muscle atrophy in this breed with negative serological tests for masticatory muscle myositis and myasthenia gravis, along with muscle biopsies suggesting an inflammatory process, support the diagnosis. However, there is an urgent need for a more specific diagnostic test. The average of inbreeding coefficient (CoI) of 16.3% suggests an increased expression of a Dog Leukocyte Antigen Class II haplotype, leading to an increased disease risk. The prognosis remains guarded, as treatment can only manage the disease. Recurrence of clinical signs and perceived poor quality of life are the most common reasons for humane euthanasia.

**Electronic supplementary material:**

The online version of this article (doi:10.1186/s12917-015-0408-7) contains supplementary material, which is available to authorized users.

## Background

The immune-mediated inflammatory myopathies are acquired diseases, characterised by immunological processes primarily involving the skeletal muscle. In human medicine these disorders are divided into five major subsets: dermatomyositis, generalised polymyositis, focal myositis, necrotizing autoimmune myositis and inclusion-body myositis [1]. All but necrotising autoimmune myositis have been described in canine medicine. Dermatomyositis affects skin and muscle and is a complement-mediated microangiopathy in which complement deposition in small vessels leads to vascular damage and muscle ischaemia [2]. Canine dermatomyositis is an autosomal dominant condition with incomplete penetrance recognised in rough collies, Shetland sheepdogs and Pembrokeshire corgis [3,4]. In generalised polymyositis and inclusion-body myositis, muscle fibres expressing antigens of the major histocompatibility complex (MHC) are infiltrated by cytotoxic T cells, leading to myofibre necrosis [2,5,6]. In contrast to man, inclusion body myositis is recognised infrequently in veterinary medicine [7]. Histopathology of inclusion body myositis is characterised by cytoplasmic filamentous inclusions, membranous structures and myeloid bodies, in addition to cellular infiltration and increased expression of MHC antigen [7].

Focal myositis is characterised by immune-mediated damage of specific muscle groups such as the masticatory muscles (e.g. temporalis, masseter, pterygoid, rostral portion of the digastricus) in masticatory muscle myositis (MMM) and the extraocular muscles in extraocular myositis. MMM is characterised by the presence of muscle-specific serum autoantibodies, most notably against masticatory myosin binding protein-C [8].

Polymyositis has been described previously in many breeds although large breed dogs are predisposed, especially Boxers, German shepherd dogs, Labrador and Golden retrievers, Doberman pinchers, and Newfoundlands [6,9-11]. A breed-specific idiopathic inflammatory polymyopathy has been described previously in dogs of the Hungarian Vizsla breed [12,13] and is characterised by cellular infiltration, particularly affecting masticatory and pharyngeal-oesophageal muscles.

The aim of this study was to describe the clinicopathological features of idiopathic inflammatory polymyopathy in the Hungarian Vizsla, in particular to emphasise the diagnostic approach, treatment, outcome, and to recommend guidelines for breeding practices.

## Results

### Signalment and clinical signs

**Figure 1:**
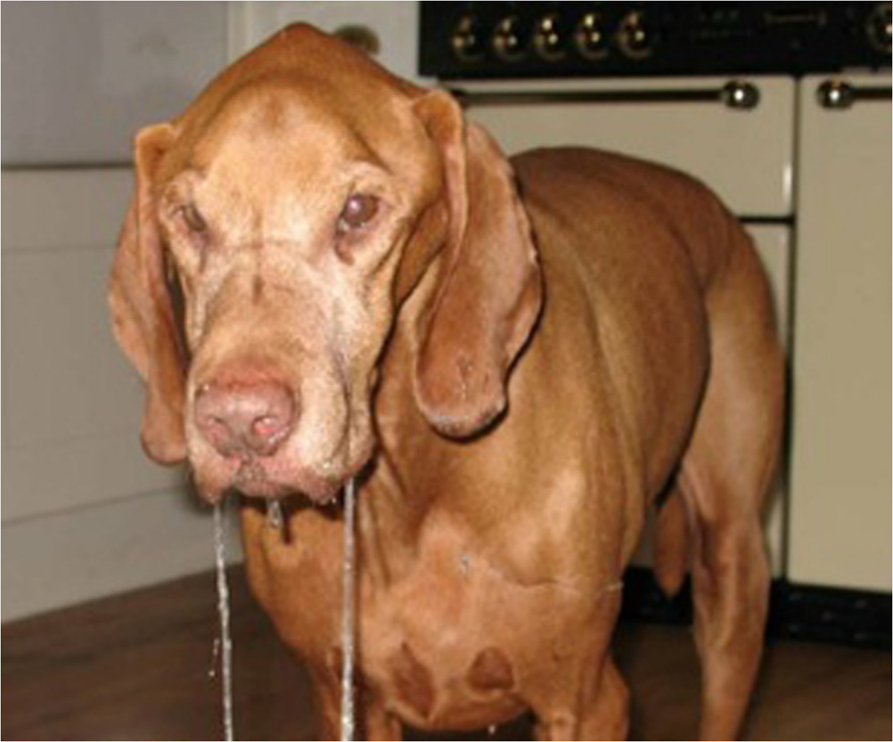
Sialorrhoea in VIP.

**Figure 2:**
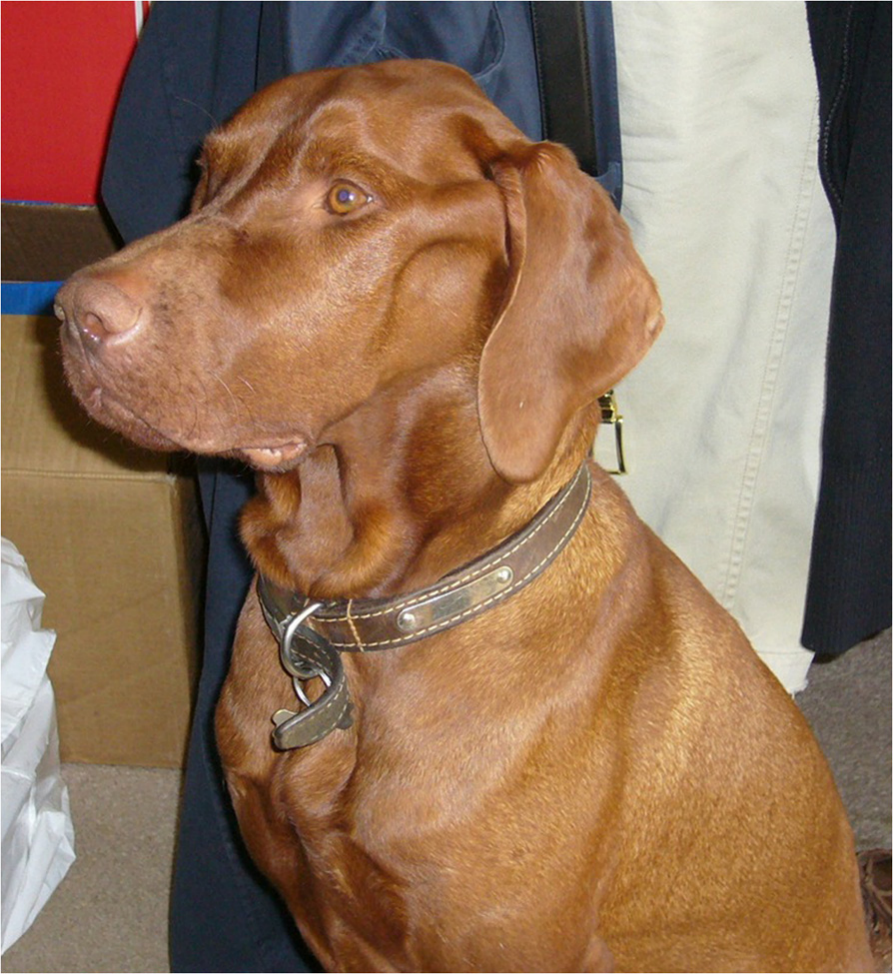
Masticatory muscle atrophy in VIP.

**Figure 3:**
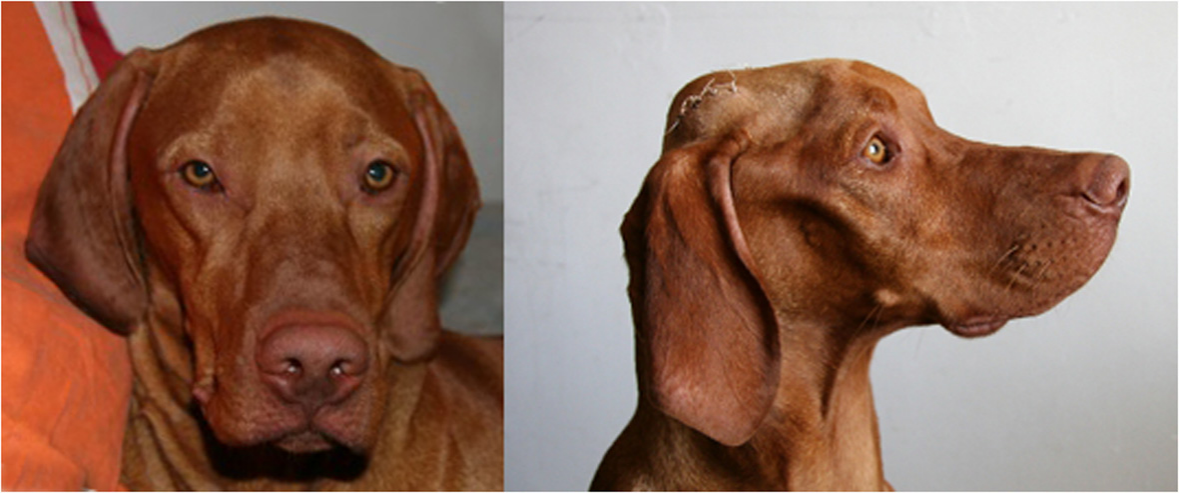
Hungarian Vizsla dog before and after VIP.

Seventy-seven of the 369 Hungarian Vizsla dogs (Tables 1 and 2) were determined to have a case history consistent with Vizsla idiopathic inflammatory polymyopathy (VIP) (Group 1, n = 24 dogs; Group 2, n = 53 dogs including eight dogs with biopsy-confirmed non-inflammatory muscle disease). Two cases were shown to have acetylcholine receptor antibodies (consistent with myasthenia gravis) and they were excluded from the study. Eight of the 77 affected cases were from outside Europe: three were from USA, one was from New Zealand, and four were from Australia. The mean age of onset was 2.4 years (range 0.2–10.3 years). Male dogs were slightly overrepresented (entire male:neutered male:entire female:neutered female 26:24:9:17). In one case the gender was unknown. The most common presenting signs in both groups (Tables 1 and 2) were dysphagia (i.e. pharyngeal phase of deglutition) (Additional file 1: Video 1) (90% of all dogs; group 1: 83% and group 2: 92%), difficulty in eating and drinking (i.e. oral phase of deglutition) (Additional file 2: Video 2) (90% combined; group 1: 87% and group 2: 91%), sialorrhea (87% of all dogs; group 1: 87% and group 2: 87%) (Figure 1) and regurgitation (79% of all dogs; group 1: 79% and group 2: 79%). Pain on opening the jaw was reported in 12% of all dogs (group 1: 4% and group 2: 15%). Masticatory muscle atrophy (i.e. masseter, temporalis and pterygoid muscles) (Figure 2) was present in 84% of all dogs (group 1: 83% and group 2: 85%), and 21% of all dogs had restricted jaw motility (group 1: 12% and group 2: 25%). Masticatory muscle atrophy either appeared early in the course of the disease, or had a more insidious onset, progressing slowly over several months or years (Figure 3). Enophthalmos, if present, was secondary to atrophy of the pterygoid muscle. Generalised muscle atrophy (43% of all dogs; group 1: 38% and Group 2: 45%), exercise intolerance (35% of all dogs; group 1: 46% and group 2: 30%), generalised weakness (30% of all dogs; group 1: 38% and group 2: 26%), and lameness (19% of all dogs; group 1: 21% and group 2: 19%) were less common signs. Three cases had dysuria (two in group 1 and one in group 2). The owners described a “stop-start flow” as if the dogs had difficulty maintaining a urine stream. The underlying pathophysiology for this was not ascertained.

**Table 1:** **Clinical and diagnostic findings in Vizsla polymyopathy**

***Clinical and Diagnostic Findings in VIP***		***Number of dogs from Group 1***	***% of total Group 1 dogs (24)***	***Number of dogs from Group 2***	***% of total Group 2 (53)***	***Total number of CASES with this clinical signs or had this diagnostic test out of total 77 dogs***	***% of total cases (77)***
*Dysphagia (pharyngeal phase of deglutition)*		*20*	***83%***	*49*	***92%***	***69***	***90%***
*Drinking and eating difficulties (Oral phase of deglutition)*		*21*	***87%***	*48*	***91%***	***69***	***90%***
*Sialorrhea*		*21*	***87%***	*46*	***87%***	***67***	***87%***
*Masticatory muscle atrophy*		*20*	***83%***	*45*	***85%***	***65***	***84%***
*Regurgitation*		*19*	***79%***	*42*	***79%***	***61***	***79%***
*Trismus*		*3*	***12%***	*13*	***25%***	***16***	***21%***
*Masticatory myalgia*		*1*	***4%***	*8*	***15%***	***9***	***12%***
*Aspiration pneumonia*		*4*	***17%***	*13*	***25%***	***17***	***22%***
*Toxoplasma gondii serumantibody titre (tested for and negative)*		*14*	***58%***	*11*	***21%***	***25***	***32%***
*Neospora caninum serum antibody titre (tested for and negative)*		*15*	***62%***	*10*	***19%***	***25***	***32%***
*Elevated serum creatine kinase (>190 IU/L)*		*18*	***75%***	*23*	*43%*	*41*	*53%*
*Serum creatine kinase >1000*		*11*	***46%***	*14*	***26%***	***25***	***32%***
*Anti- 2 M antibody titre (Tested for and negative)*		*12*	***50%***	*17*	***32%***	***29***	***38%***
*Anti-acetylcholine receptor antibody titre (Tested for and negative)*		*16*	***67%***	*16*	***30%***	***32***	***42%***
*Electromyography (Performed and findings suggestive of myopathy)*		*15*	***62%***	*5*	***9%***	***20***	***26%***
*Electromyography (Performed and findings normal)*		*1*	***4%***	*2*	***4%***	***3***	***4%***
*Modality by which diagnosis Megaoesophagus was made*	*Radiographs*	*8*	***33%***	*18*	***34%***	***26***	***34%***
	*Barium*	*1*	***4%***	*9*	***17%***	***10***	***13%***
	*Fluoroscopy*	*4*	***17%***	*8*	***15%***	***12***	***16%***

**Table 2:** **Clinical signs of idiopathic inflammatory polymyopathy in the Vizsla – Percentage of dogs with this clinical sign is indicated (both Group 1 and 2 combined)**

**Clinical signs in VIP**			
**Most common**		**Less common**	
**Dysphagia (pharyngeal phase)**	90%	**Other muscle atrophy**	43%
**Drinking/Eating difficulty (oral phase)**	90%	**Exercise intolerance**	35%
**Sialorrhea**	87%	**Weakness**	30%
**Masticatory muscle atrophy**	84%	**Trismus**	21%
**Regurgitation**	79%	**Lameness**	19%
		**Masticatory myalgia**	12%

In addition to clinical signs of neuromuscular disease, 25 dogs (seven in group 1, 18 in group 2) had other comorbidities either concurrently or at some other stage in their life, including other inflammatory diseases (Table 3): 17 dogs with atopic dermatitis; two dogs with immune-mediated polyarthritis; nine dogs with inflammatory bowel disease; three dogs with keratoconjunctivitis sicca; one dog with sebaceous adenitis; one dog with steroid-responsive meningitis arteritis. There were also three dogs with idiopathic epilepsy, one dog with a fly-catching repetitive behavioural disorder and one dog with splenic haemangiosarcoma. Fourty dogs were dead at the time of writing and dysphagia and aspiration pneumonia were reported to be the main cause of death.

**Table 3:** **Other idiopathic immune-mediated diseases seen in the dogs in this series**

**Concurrent immune-mediated diseases reported in the dogs in this series with VIP**	**Number of dogs**
Atopic dermatitis	**17**
Immune-mediated polyarthritis (IMP)	**2**
Inflammatory bowel disease (IBD)	**9**
Keratoconjunctivitis sicca	**3**
Sebaceous adenitis (SA)	**1**
Steroid-responsive meningitis arteritis (SRMA)	**1**

### Laboratory findings

**Figure 4:**
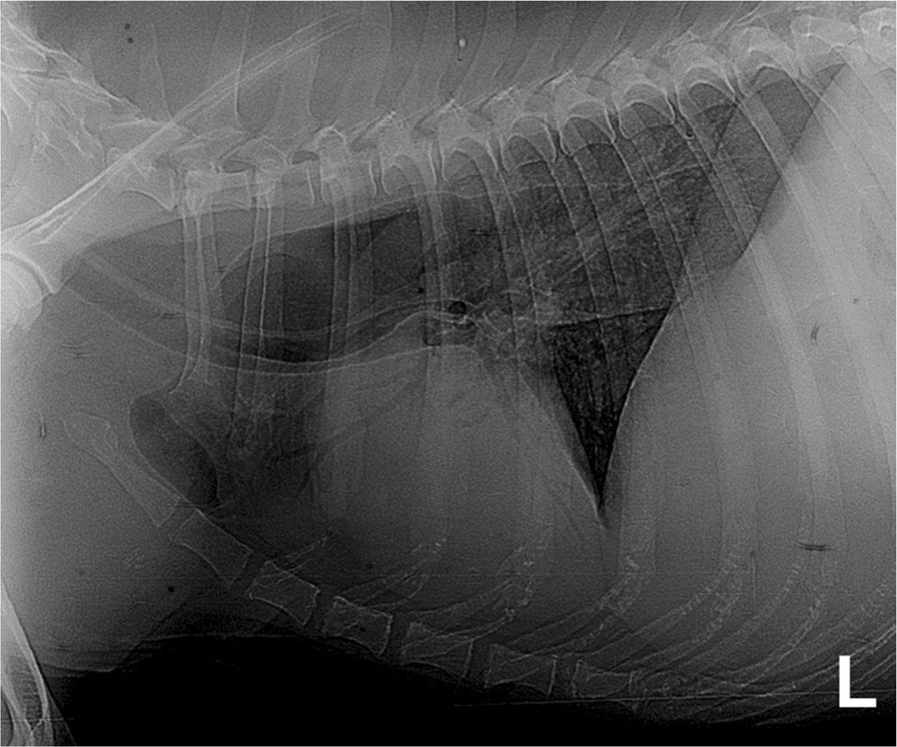
Left lateral thoracic view, showing megaoesophagus.

**Figure 5:**
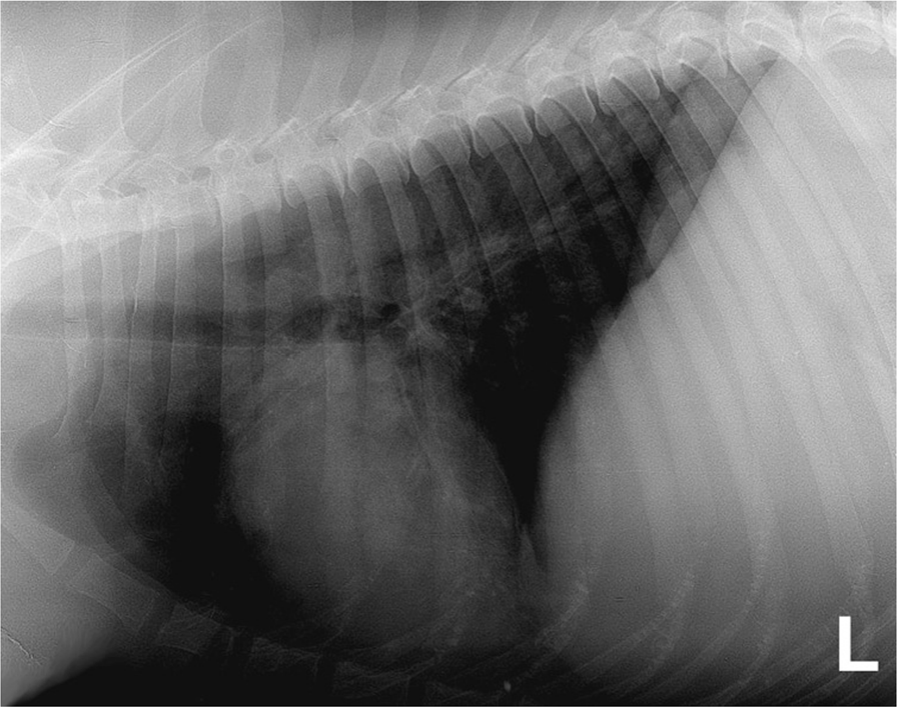
Left lateral thoracic view, showing aspiration pneumonia.

**Figure 6:**
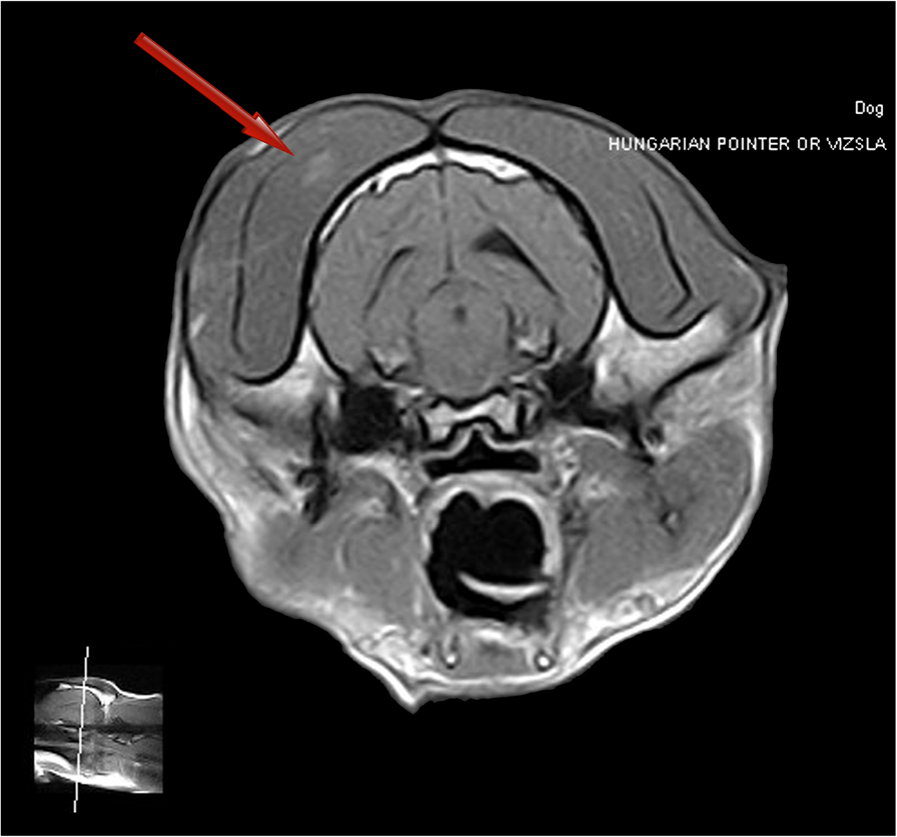
Transverse T1–weighted post-gadolinium contrast image at the level of the optic chiasm. Patchy up take of contrast is present within the right temporal muscle (arrow) suggesting an inflammatory process.

A summary of the diagnostic tests performed in each dog is detailed in Table 1 and more detailed results are available in Additional file 3. Serum CK activity was evaluated in 47/77 of cases and it was elevated (>190 IU/L) in 87% and above 1000 IU/L in 53%. In four cases the result was unknown. Serology for determination of antibodies against 2 M fibres for masticatory muscle myositis (MMM) or for antibodies against acetylcholine receptors for MG was performed in 29 and 32 of 77 dogs, respectively. Serology for MMM was negative in all cases tested; two dogs were positive for MG and were excluded from this study. Serology for protozoal diseases causing inflammatory myopathy (*Toxoplasma gondii* and *Neospora caninum* serum antibody titres) was negative in 25 cases. Imaging techniques were performed in 52 cases. As part of the diagnostic work up for regurgitation, 28/77 of the dogs had oesophagogastroduodenoscopy. This investigation revealed the presence of *Helicobacter* spp. in two dogs and biopsy evidence suggestive of inflammatory bowel disease (IBD) in nine dogs. Thoracic radiographs confirmed megaoesophagus in 28 dogs (Figure 4) and aspiration pneumonia in 17 dogs (Figure 5). A barium study was performed in 10 cases; however, this test was not superior to plain radiography in proving oesophageal dysfunction. Fluoroscopy was performed in 12 cases where routine radiography with or without barium had not proved oesophageal dysfunction and in three dogs dysmotility, mainly involving the oral and pharyngeal phase, was demonstrated. Electromyography (EMG) of the appendicular and axial muscles including the masticatory muscles showed generalised abnormal spontaneous activity in 20 of 23 dogs, including positive sharp waves, fibrillation potentials, prolonged insertional activity, and occasional pseudomyotonia. EMG also confirmed tongue and pharyngeal involvement in 11 of 23 dogs. Magnetic Resonance Imaging (MRI) of the head was performed in five cases, and in two dogs it revealed changes within the masticatory muscle consistent with a multifocal inflammatory process. Identification of this change suggested a suitable biopsy site where clinical examination and other diagnostic tests such as EMG had not confirmed a diagnosis or the focal location of the pathology (Figure 6).

**Figure 7:**
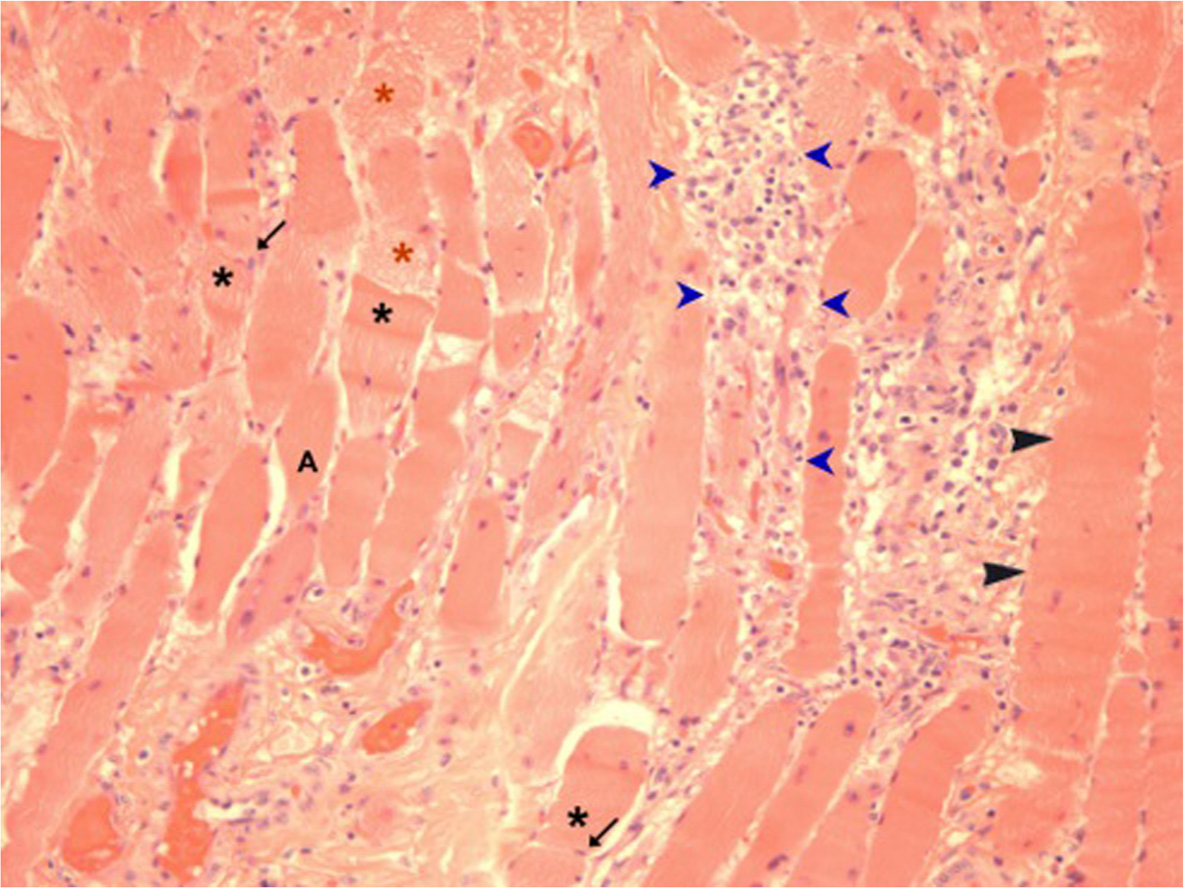
Histopathological section of temporal muscle (H&E stain, x100). Mild variation of myofibre size, with internal nuclei (black arrow) found along the plane of the fibre splitting (star), hypertrophied myofibres (black arrow head), granular sarcoplasm (yellow star), angular atrophy (A), and necrosis with infiltration of inflammatory cells (blue arrow head).

Muscle biopsy samples were taken in 36 of the 77 cases (37%) (Table 4); however, in seven cases the biopsy reports were not included in the case notes. In twenty-five of the 29 dogs for which histology was available, the biopsies were taken from the most accessible sites such as masseter, temporalis, lingual, triceps and cranial tibialis muscles, while in four cases the biopsy location was not reported. In 20 of the 25 dogs underwent biopsy following EMG or MRI identification of the most potentially useful sites. Post-mortem examinations were performed in four of the 40 deceased dogs and besides being a valuable aid towards confirming the diagnosis with multifocal lymphoplasmacytic and macrophagic cellular infiltrations having an endomysial and perimysial distribution with invasion of non-necrotic fibres being the most common histopathological finding in both ante and post mortem samples. In addition, in one case post-mortem examination revealed the presence of lymphoid cell infiltrate in the oesophageal myenteric plexus with diminution in the number of ganglion cells (Table 3). Degenerative (e.g. variation in myofibre size, myofibre hyalinisation, necrosis, angular atrophy, nuclear internalisation, granular sarcoplasm, sarcolemmal fragmentation) and regenerative changes (e.g. cytoplasmic basophilia, nuclear rowing, presence of type 2C fibres, compensatory hypertrophy) were found variably (Figure 7). Myofibre loss, endomysial fibrosis and excessive perimysial fatty tissue were also found (Table 4). The presence of the adipose tissue was considered likely to be secondary to chronic injury as fat infiltration has been reported in chronically denervated muscles, but may also occur in severe chronic degenerative myopathy [14]. In 7/32 cases there was an absence of inflammatory changes. This was probably associated with recent steroid therapy (in two cases) or end-stage disease. However, in three dogs with recent onset disease that had not been treated, there was a degenerative and regenerative myopathy in the absence of obvious inflammatory change. In two cases antibodies against endplate proteins (SPA-HRPO) were identified.

**Table 4:** **Histological changes in the 32 Hungarian Vizslas with biopsy-confirmed polymyopathy**

***Histopathological changes (Total 32)***	***Group 1***	***Group 2***
	***25 total dogs (biopsies) (confirmed VIP diagnosis)***	***7 dogs (3 biopsies, 1 biopsy and post-mortem/3 post-mortem) (myopathy diagnosis)***
*Inflammatory change e.g. Lymphohistiocytic inflammation*	Variability in myofibres size with multifocal endomysial, interstitial and perivascular mononuclear cell infiltrations (lymphocytes & macrophages +/− plasma cells, eosinophils) of non-necrotic fibres. Underlying inflammatory process masked by corticosteroid treatment in one case.	
	25 dogs	0 dogs
*Myopathic change*		Variation in the myofibre size without inflammatory infiltration.
	0 dogs	7 dogs
*Adipose tissue*	Small amount of adipose tissue associated with fibrosis.	Adipocytes present in some fascicles (endomysium and perimysium).
	2 dogs	1 dogs
Fibrosis	None OR perimisial/endomysial fibrosis OR occasionally area of fibrosis with lack of myofibres with any significant inflammation (primary or secondary?)	
	3 dogs	0 dogs
Degenerative changes	Either any appreciable myofibre degeneration or active degenerative changes within the muscle fibres (variation in myofibre diameter, atrophy with round to polygonal/angular shape, hyalinisation, nuclear internalisation, sarcolemmal fragmentation).	Variation in muscle fibres, atrophy (occasionally smaller fibres grouped together, some angular but most round to polygonal profile), nuclear internalisation.
	19 dogs	4 dogs
Regenerative changes	Nuclear rowing, centralisation/hyperthrophy of the nuclei increased cytoplasmic basophilia, type 2 fibres.	Occasional enlarged and round myofibres (compensatory hypertrophy), and nuclear rowing.
	13 dogs	3 dogs
Necrotic fibres	Scattered to severe necrotic myofibres with some undergoing phagocytosis.	
	7 dogs	0 dogs
Fibrosis	Mild to moderate endomysial and perimysial fibrosis secondary to myofibre loss.	Increased endomysial and perimysial connective tissue secondary to myofibre loss.
	7 dogs	2 dogs
Intramuscular nerve branches	Normal (25 dogs)	Normal (7 dogs)
Immunoreagent SPA-HRPO	Present in two cases	
(Antibodies against endplate proteins)		
Dystrophin protein		Decreased staining for carboxy terminus of the dystrophin protein was found in one case; however, the dog improved on immunosuppressive treatment and the suspicion for muscle dystrophy was abandoned.

### Management

Sixty-one of 77 of the cases were treated with immune-suppressive doses of corticosteroids either as a monotherapy (32/61) or in combination with other immunosuppressive treatments (29/61). For the other 16 of 77 dogs, no treatment details were stated in the clinical record. A combination of immunosuppressive agents was considered preferable to reduce long-term corticosteroid side effects and/or when the clinical response to monotherapy was poor. The most common polypharmacy was prednisolone and azathioprine (25/29). Less commonly, ciclosporin was used in combination with prednisolone (2/29) or with both prednisolone and azathioprine (2/29). Leflunomide was used as an adjunct drug in one case in association with corticosteroids. Two cases were treated with methotrexate, either with corticosteroids or in combination with corticosteroids and azathioprine. The typical initial glucocorticoid dose was 1-2 mg/kg prednisolone twice a day, whilst azathioprine was used at 2 mg/kg or 50 mg/m^2^ once a day. Leflunomide was used at 4 mg/kg once a day, while methotrexate was given at 2.5 mg/m^2^ twice a week. The dosages were then tapered based on the response to treatment. Improvement of clinical signs especially sialorrhea, regurgitation, dysphagia was seen in 90% of the treated dogs. Unfortunately, we were unable to evaluate time frame to improvement due to lack of detailed temporal data, however the use of anti-inflammatory dose of corticosteroids or a rapid tapering regimen with withdrawal of drugs within a 1-year period appeared to be associated with earlier relapse and increased mortality in 23% of the treated cases. Two dogs treated with azathioprine had adverse gastrointestinal effects (i.e. profound vomiting and occasional diarrhoea) and exhibited marked elevation in serum hepatocellular enzymes resulting in cessation of therapy. Ciclosporin was discontinued in one case due to severe inappetence, while the other three cases in which this drug was used had undetermined clinical benefit. Corticosteroid side effects such polyphagia, polydipsia, polyuria, and iatrogenic hyperadrenocorticism were found in 15% of the treated cases. Supportive treatments including gastroprotectants and pro-kinetics were also commonly prescribed such as omeprazole, sucralfate, cimetidine, famotidine, ranitidine, maropitant, metoclopramide, erythromycin (as a pro-kinetic at a dose of 0.5-1 mg/kg three times a day), and cisapride. Three cases were treated with phenobarbital on the initial assumption that their diagnosis was phenobarbital-responsive hypersalivation, but there was no clinical benefit reported. In the dogs with dysuria, one dog was treated with 0.35 mg/kg diazepam three times a day and the other with 1 mg/kg dantrolene twice a day and 0.4 mg/kg phenoxybenzamine twice a day, and both showed clinical improvement; the third dog was treated with phenylpropanolamine at the dose of 0.8 mg/kg once a day and diazepam at the dose of 0.25 mg/kg twice a day with no improvements, while corticosteroid therapy was reported to be beneficial.

At the time of writing, 40 of 77 affected cases had died: thirty-seven dogs were euthanised due to the disease and three died of other causes including haemangiosarcoma, idiopathic epilepsy and reported natural causes. The mean age of death was 6.4 years (range 1.0 – 14.5 years), and the mean survival time following diagnosis was 3.9 years (range 0.1 – 12.5 years). Recurrent aspiration pneumonia and poor quality of life because of difficulty eating and drinking were common reasons cited for euthanasia.

## Discussion

This paper describes a myopathy in the Hungarian Vizsla characterised by dysphagia, regurgitation, sialorrhea and masticatory muscle atrophy, which is responsive to immunosuppression in the majority of cases. Biopsy sampling in a proportion of cases suggested an idiopathic inflammatory polymyopathy. One of the authors (CR) first diagnosed idiopathic inflammatory polymyopathy in a Hungarian Vizsla in 1994, but anecdotal reporting by owners suggests that the disease has been a problem in the breed since the early 1980s or before. A serious shortcoming in this retrospective study was the inclusion of non-biopsy confirmed cases. However, group 2 dogs were included because excluding them might give the erroneous impression that this syndrome can be easily diagnosed by biopsy and exclusion of these cases would also lose clinical data that might be valuable to practising clinicians. Moreover, it is possible that this syndrome might actually represent a collection of immune-mediated diseases in the Hungarian Vizsla with a common theme of dysphagia, regurgitation and masticatory muscle atrophy; so although biopsy remains the gold standard for diagnosis, it was not always useful and in seven cases the muscle biopsies did not confirm an inflammatory process despite using EMG in two of these cases to direct biopsy and the dogs subsequently responding to immunosuppressive therapy. Post-mortem was performed in four of these seven cases with suspicion of VIP and in two of them the disease was confirmed. This finding, and the differences between VIP and the classical description of canine polymyositis [9] (in particular dysphagia), raises the question as to whether the pathogenesis is different. Diagnosis of typical myositis in man is dependent on the presence of inflammatory infiltrates and positive human leukocyte antigen (HLA – ABC) labelling of the sarcolemma. However, other immune-mediated myopathies exist, e.g. human necrotising myopathy has no inflammatory infiltrates which is associated with anti-signal recognition particle autoantibodies. Dysphagia is an important clinical feature of this disease and patients respond to immunosuppression [15]. A future goal to improve diagnosis of VIP is to investigate whether or not it is possible to identify myositis-specific autoantibodies. In the present study, incubation with staphylococcal protein A conjugated to horseradish peroxidase (SPA-HRPO) detected antibody (IgG) bound to the neuromuscular junction in two group 1 cases. This labelling was suggestive of an autoimmune polymyositis. In the present study, nine out of 29 muscle biopsies were subjected to labelling with SPA-HRPO (not used in three cases and not reported in the remainder) and further studies are necessary in order to better understand the pathophysiology of this disease and to aid the diagnosis. However, until this data is available, the following recommendations are offered to maximise the chance of successful diagnosis: (1) biopsy of end-stage muscles should be avoided as adipose or connective tissue replaces muscle tissue and diagnosis may be challenging, so clinicians should chose to biopsy muscles which are not severely atrophied); (2) our data suggests the most useful muscle biopsy site is the temporal muscle, which was sampled in 22/29 (76%) of the present cases and confirmed diagnosis in 19 cases; (3) lingual muscle biopsy was performed in five cases for which biopsy samples were collected and in two of these the procedure assisted in making a diagnosis, so this may also be useful biopsy site and this procedure is especially indicated when dogs are presented with oropharyngeal dysphagia; (4) EMG and MRI were valuable in helping identify the appropriate muscle to sample (Figure 6).

EMG has lower cost, but may not detect mild disease especially if there is minimal involvement of the appendicular muscle. MRI is generally more expensive to perform but where performed showed good association with the histopathology results. In people, whole-body MR imaging using rapidly acquired fat-suppressed Short TI Inversion Recovery (STIR) sequences has been recommended to facilitate a global overview of the extent and symmetry of muscle disease and to direct biopsy collection. STIR signal intensity has been documented to have a 97% specificity for identifying sites of inflammatory myopathy that have been confirmed at biopsy [16] [although, where previous corticosteroid treatment is used, post-contrast images may be more helpful. Inflammatory muscle lesions on MR images were characterised by diffuse and poorly marginated abnormal signal on T1- and T2-weighted images, with marked enhancement after contrast medium administration [16].

Other laboratory tests can be used to support a diagnosis of VIP and rule out other diseases, as when making a diagnosis of VIP, other causes of myopathy should be ruled out. The most important differential diagnoses are toxins [17], endocrine disease [18-20] and infectious causes of inflammatory myopathy, especially Toxoplasma gondii, Neospora caninum and in some geographical regions Leishmania, Ehrlichia canis, Sarcocystis neurona and Hepatozoon americanum. A limitation of our study was the lack of completeness of such laboratory tests for many cases. However it is noted that dysphagia and/or regurgitation are rarely reported as clinical signs of inflammatory myopathy associated with infectious diseases. In a comprehensive literature search [21-45] a single case report was found describing dysphagia and regurgitation in a puppy with neonatal neosporosis that also presented with neuromuscular paralysis [45]. It is not unusual for neospora tachyzoites to be found in the oesophagus on histological examination of post mortem material but more uncommon for there to be associated clinical signs and therefore the clinical significance is uncertain [35,38]. Therefore, although infectious diseases should be ruled out as an underlying cause in cases of suspected VIP, it is a rare differential for adult dogs presenting with dysphagia and regurgitation. Given the clinical presentation of VIP, the most important differential diagnoses are MMM and MG, which may also show similar clinical features to VIP. Thus, serum titres for antibodies against type 2 M fibres and the acetylcholine receptor should be evaluated. Occasionally a dog may present with both myasthenia gravis and VIP [46]. In our study MMM was not diagnosed in any of the affected dogs, although it should be noted that only 38% of dogs were tested.

Marked elevation of CK is an indication of damage to skeletal muscles; however, only 25 of the 47 dogs tested had an elevation above 1000 IU/L. This may be because generalised muscle disease was not a feature. In most cases of VIP, the masticatory and pharyngeal muscles were most severely affected and constituted only a small volume of muscle mass compared with the whole body. Of the 25 dogs with marked elevation of CK, 18 had generalised muscle atrophy and/or exercise intolerance, but seven were considered to be normal in this regard.

The most consistent clinical signs of VIP are dysphagia and sialorrhea due to tongue, pharyngeal and oesophageal dysfunction, and masticatory muscle atrophy. The canine oesophageal anatomy differs from other species such as cats and people, especially in the musculature of the oesophageal body. In dogs the cranial oesophageal (cricopharyngeal) sphincter and the entire oesophageal body is composed of striated muscle, while the caudal oesophageal (gastroesophageal) sphincter is smooth muscle. Therefore the canine oesophagus is often involved in diseases that affect skeletal muscle [47]. However, in at least two of the present series of cases there were alternative explanations for the oesophageal involvement identified on post-mortem examination: in one case there was no primary inflammatory process in oesophageal muscle, but there was invasion of inflammatory cells secondary to degeneration of myocytes and a second case revealed lymphoid cell infiltration of the oesophageal myenteric plexus with diminution in the number of ganglion cells, which may represent a ganglionopathy. The latter histopathological picture is similar to that observed in oesophageal achalasia in man [48]. Oesophageal achalasia is characterised by dysfunction of the lower oesophageal sphincter and derangement of oesophageal peristalsis. The cause is not fully known, but autoimmune processes appear to be involved in human patients with a genetic susceptibility to the disease [48].

The dysphagia reported in this case series may not be entirely due to oesophageal dysfunction, as masticatory, pharyngeal, and lingual muscle involvement could contribute to difficulty eating. In this case series, atrophy of the masticatory muscles was a common feature of the disease. Polymyositis with masticatory muscle atrophy and tongue involvement has been recognized previously in Pembroke corgi dogs in which end-stage lingual atrophy was a predominant feature [49]. In the present series, atrophy of the tongue was observed as an end-stage of disease in three untreated dogs and in one clinical history, the tongue was described as being shrivelled, soft, withered and dry with deficits of sensation as well as motor function. Sensory deficits could not be confirmed, however, the tongue is richly innervated and it is theoretically possible that end-stage fibrosis might interfere with nervous supply.

Perhaps surprisingly for a polymyopathy, exercise intolerance was not the most common clinical sign, with normal exercise ability being reported in 27 of 77 dogs and only 33 of 77 dogs developed generalised muscle atrophy. Others have described the clinical presentation of canine polymyositis being characterised by cervical ventroflexion and dysbasia (a stiff gait with short stride a.k.a. “walking on eggshells”); [9]. This was not a feature of VIP. The lack of appendicular muscle involvement coupled with an insidious onset of the disease may be a reason for delayed or incorrect diagnosis.

Dysuria was a surprising clinical sign associated with a skeletal muscle disease but was reported in three male dogs with biopsy-confirmed VIP. The detrusor is smooth muscle, so if detrusor myopathy was a cause of the dysuria then it would imply the presence of autoantibodies against smooth as well as skeletal muscle, which would be unlikely as in all human and laboratory animal models of myositis, autoantibodies are specific to striated muscle and correlate with distinct clinical phenotypes [50,51]. Additionally, an immune-mediated process targeting smooth muscle would reasonably be expected to result in intestinal and arterial smooth muscle disease too. Immune-mediated damage of the skeletal muscle of the external urethral sphincter is possible, although this might have been expected to result in clinical signs of sphincter mechanism incompetence. The possibility of a neurological cause of dysuria cannot be excluded; however, an anatomical difference exists between the urethra of female and male dogs, which may explain why dysuria was present only in male dogs. Female urethral smooth muscle occupies one-third of the volume of the vesical neck (both the bladder neck and the urethral smooth muscle constitute the internal urethral sphincter) and one fourth of the volume of the proximal urethra, while striated muscle is present in the distal half of the urethra [52]. In contrast, the male urethral smooth muscle is associated mainly with the trabeculae surrounding the prostate lobules and is fundamental for contraction of the lobules (i.e. the bladder neck acts as smooth muscle sphincter), while striated muscle is present caudal to the prostate (post-prostatic urethra), which also overlaps the caudal surface of the prostate gland [53]. Although we need to consider that in this study fifty of the 77 affected cases were male dogs, it can be speculated that the dysuria in male dogs with VIP may be associated with more extensive involvement of urethral striated muscle.

An easier and more reliable method of detecting canine oesophageal dysmotility would be of great benefit because complications associated with dysphagia and aspiration pneumonia are the main cause of death in VIP. Deglutition consists of three phases: the oral phase that occurs in the mouth and involves lips, tongue, teeth, and palate, and it is essential for the formation of the food bolus; the pharyngeal phase that is essential for the progression of the food bolus from the mouth to the oesophagus and preventing entry into the airway and the oesophageal phase that allows the food bolus to travel through the oesophagus towards the stomach. Plain or contrast thoracic radiographs may reveal megaoesophagus; however, if less severe or dynamic disease of the oesophagus is present, fluoroscopy may be more useful, especially in the detection of swallowing disorders that mainly involve the oral and pharyngeal phase. However difficulties in performing this test were reported either due to lack of equipment or to the difficultly in keeping the animal steady for the image intensifier. It is also advisable to perform these studies with great care in order to avoid barium aspiration pneumonia. In man, oesophageal manometry is considered the ‘gold standard’ for assessing oesophageal motor function and is used to measure the pressures and the pattern of muscle contractions in the oesophagus, and to detect abnormalities in the contractions and strength of the oesophageal muscle and its sphincter [54]. Over the last few years intraluminal oesophageal manometry has developed into high-resolution manometry, which improves acquisition of data, and the method of displaying and analysing the data using oesophageal pressure topography plots. This new technology is also patient-friendly as it allows for a shorter procedure time, and it is much easier to use and to interpret compared with conventional manometry [55]. High-resolution manometry could be useful in the detection of oesophageal disorders in animals, but further studies are required to confirm this. Previously the majority of the veterinary studies have used conventional manometry, and most of them used anaesthetised dogs [56-61].

The presence of *Helicobacter* spp. in two dogs and inflammatory bowel disease (IBD) in eight dogs that underwent oesophagogastroduodenoscopy was considered incidental to the primary diagnosis of VIP. *Helicobacter* spp. have been associated with chronic superficial gastritis in man and play a role in the pathogenesis of peptic ulcer disease, gastric carcinoma and lymphoma, but their clinical significance in companion animals is not clear [62]. The finding of IBD could be more significant, especially when the breed appears predisposed to other immune-mediated diseases including MG, MMM, atopy, sebaceous adenitis, keratoconjunctivitis sicca, steroid-responsive meningitis arteritis, and immune-mediated polyarthritis (Table 2). Vizslas have been also reported anecdotally to be predisposed to immune-mediated haemolytic anaemia [63] and immune-mediated thrombocytopenia [64]. There are occasional reports of human patients with both IBD and polymyositis [65]. The disruption of the tight junctions between enterocytes plays an important role in the pathophysiology of IBD, altering the intestinal epithelial barrier function and causing increased intestinal permeability. The ‘leaky gut’ may then lead to translocation of bacteria and endotoxin, which drives the intestinal inflammation. A normal response from the immune system should deal with the insult. However, in some individuals this normal response may be defective, increasing the risk of developing IBD, coeliac disease and possibly myositis [65]. When investigating VIP, we therefore recommend that comorbidities are looked for and treated if identified.

VIP is assumed to be an autoimmune disease because of the response to immunosuppressive treatment. The cornerstone of the treatment is glucocorticoid therapy due to its short-term tolerability, cost effectiveness, and clinical effectiveness [66]. Particular care however must be taken if there is a risk of aspiration pneumonia as the adverse effects of polydipsia, polyphagia and immunosuppression increases the likelihood of this potentially fatal complication. As the individual treatment plans for the dogs in this study varied, it was not possible to make firm conclusions regarding treatment, but the trends suggested the following: (1) early diagnosis improves the chance of successful treatment; (2) therapy should be tapered slowly with time of tapering depending on clinical signs. We do not have enough data to strongly determine the dosage and the length of the treatment protocol for the VIP, however inadequate corticosteroid dosage i.e. anti-inflammatory dose or a rapid tapering regimen with withdrawal of drugs within a 1-year period appeared to be associated with earlier relapse and increased mortality in 23% of the treated cases. The optimal glucocorticoid dosing regimen is not well defined [13]; however, as a general rule we would suggest the following: 2 mg/kg once a day for 2 weeks, then 1 mg/kg once a day for 4 weeks, then 0.5 mg/kg once a day for 10 weeks, then 0.25 mg/kg once a day for 16 weeks, then 0.25 mg/kg every other day for 16 weeks then continue to withdraw over a further 2–4 weeks; (3) close monitoring is required and the drugs are tapered on the basis of clinical signs and the concentration of serum CK, if this test had shown abnormal result prior treatment.

In four dogs, dysphagia and regurgitation prevented the use of oral therapy, but there was a good response to initial parenteral short-term corticosteroid therapy such as with dexamethasone and prednisone acetate. Other parental corticosteroids such as methylprednisolone seemed to be less effective (two cases), however the difficulty of comparing different treatments used is a limitation of this paper and further study is needed to establish the most effective therapeutic protocol for this disease. This study also suggested that a treatment based on a combination of immunosuppressive agents was preferable to reduce long-term corticosteroid side effects such polyphagia, polydipsia, polyuria, and iatrogenic hyperadrenocorticism, reduce the risk of aspiration pneumonia and/or when the clinical response to monotherapy was poor. The most common and successful drug used in addition to corticosteroids was azathioprine; however a well-designed prospective trial needs to be performed evaluating different treatments. The dosage of azathioprine used but we would suggest 2 mg/kg once a day for up to ten days, then 2 mg/kg every other day thereafter. Although our study cannot assess the most effective treatment protocol, we would advise that this dosage should be maintained for a month beyond the cessation of prednisolone. Again this regimen should be adjusted on the basis of assessment of both clinical response and serum CK levels. To reduce the risk of aspiration pneumonia and to aid swallowing dogs should be fed from a height and with small meals 4–6 times daily. Some foods are easier to swallow than others and our experience is that individually feeding small walnut-size balls of firm, but slippery-textured commercial food with a high protein and fat content is the most useful. This should be coupled with coupage after feeding in order to encourage belching and help to prevent aspiration. In some cases an anti-gulping bowl (Dogit® Go Slow Anti-Gulping Dog Bowl, Rolf C. Hagen Ltd., West Yorkshire, UK) can be useful, especially in dogs with polyphagia due to high dosage of corticosteroids. In cases where the megaoesophagus impedes the propulsion of the food bolus, we recommend the use of the Bailey chair in order to maintain a truly vertical oesophageal position and reduce the risk of aspiration pneumonia.

Immune-mediated diseases are thought to develop through a complex combination of genetic and environmental factors and have been associated with variants of MHC class I or II genes, which in the dog are referred to as the dog leukocyte antigen (DLA) system. The MHC molecules control the immune system’s recognition of self and non-self-antigens. Class II molecules are involved in presenting antigens to CD4^+^ T cells (T helper cells) [67]. CD4^+^ T cells play a key role in regulating immune system function with a subset of immunoregulatory cells (CD25^+^CD4^+^) suppressing proliferation of other immune cells, especially CD25^−^CD4^+^ T cells and CD8^+^ T cells [68]. The basis of autoimmune reactions is the failure of this immunoregulatory T cell to properly control other immune cells and down-regulate the immune response. In polymyositis, inflammatory cells, including T cells and macrophages, are concentrated in the endomysium and surround and invade non-necrotic fibres [6]. Classically there are more CD8^+^ T cells than CD4^+^ T cells; however, Haley and others reported that cellular infiltrates in two dogs with VIP were composed predominantly of CD4^+^T cells with fewer CD8^+^T cells [13]. Labelling for MHC class I and class II antigens was increased on the sarcolemma and on the membrane of infiltrating cells in Haley and others study, however the assessment of this in our biopsies was not undertaken and needs to be considered in future studies to further help elucidate the pathogenesis of VIP.

Genes within the MHC are unusual because they are highly polymorphic, meaning that there are many allelic variations. This degree of variation most likely improves survival against infectious diseases. However in the dog, selective inbreeding has led to a restriction of DLA haplotypes in many breeds, which in turn influences susceptibility to infectious diseases and also to immune-mediated conditions [69]. Our group investigated DLA class II associations from 212 Hungarian Vizsla dogs, which were stratified both on disease status and degree of relatedness to an affected dog. One haplotype, DLA-DRB1*02001/DQA1*00401/DQB1*01303, had a significantly raised frequency in cases compared with controls. A single copy of the risk haplotype was sufficient to increase disease risk, with the risk substantially increasing for homozygotes. There was a trend of increasing frequency of this haplotype with degree of relatedness, indicating a low disease penetrance and suggesting involvement of other genetic and environmental factors [70]. Further genetic studies are required; however, our immediate advice to breeders wishing to reduce the risk of VIP is that a bitch should only be mated to a dog when the inbreeding coefficient (CoI) of the resulting puppies, as measured from a five generation pedigree, is less than 12.5% [71]. The CoI is the probability of homozygosity by descent, and it ranges from 0% to 100%. In other words, the lower the inbreeding coefficient, the lower the probability of homozygosity with a CoI of over 25% being the equivalent of a mother/son mating. The mean CoI in Vizsla Breed is 5.1% [72, 73]; however, the average CoI of 77 of the 79 dogs in our study of Vizslas was 16.3% (range: 2.5% - 40.7%). However it should be remembered that having a low CoI will not protect against VIP and breeding of dogs with immediate relatives with VIP should be avoided.

## Conclusions

The Hungarian Vizsla has an inherited predisposition to a form of inflammatory polymyopathy. The most common clinical signs are dysphagia, sialorrhea, masticatory muscle atrophy, and regurgitation. The mainstay of treatment is immunosuppressive therapy in addition to feeding therapy designed to meet individual needs. Early diagnosis, careful monitoring and slow withdrawal of medication improves prognosis. To improve diagnosis the feasibility of other diagnostics techniques such as a high resolution manometry should be investigated. Further genetic and immunological studies will better define VIP; however, until then reducing inbreeding in order to minimise homozygosity of the risk haplotype is recommended.

## Methods

Our retrospective cohort study was based on 369 medical records of Hungarian Vizsla dogs. The records were collected from dogs from which DNA had been submitted to the Centre for Integrated Genomic Medical Research (CIGMR, University of Manchester, UK) between 1992 and 2013. Cases had been recruited following a nationwide appeal for Hungarian Vizslas affected with polymyositis and their affected or unaffected relatives [74-76]. Details of the phenotype were generated for each case [77], stating Kennel Club registration number, pedigree name, common name, coat colour, gender, age, and weight. It also indicated clinical signs, diagnostic tests performed, and treatment. Based on certainty of diagnosis of VIP the cases were divided into two groups. Inclusion criteria for Group 1 were clinical signs and histopathology findings compatible with an inflammatory polymyopathy. Inclusion criteria for Group 2 was clinical signs of neuromuscular disease including presence of dysphagia, sialorrhea, temporal muscle atrophy, elevated serum creatine kinase (CK) activity, and sufficient clinical history to suggest that other neuromuscular disorders could be ruled out. This group also included dogs where muscle biopsy or post-mortem had been performed confirming a myopathy, but with no evidence of an inflammatory cell infiltrate. Pedigrees from dogs of all of the affected families were researched and collated, and a pedigree database was created. In order to investigate the genetic basis of this disorder, DNA was extracted from blood in excess of that required for diagnostic tests. Alternatively, oral swabs were submitted to the CIGMR in order to investigate the genetic basis of this disorder [70].

This is a non-experimental study based on a retrospective analysis of necessary diagnostic results and clinical history for which there was full owner written consent.

## Additional files

Additional file 1: Video 1. Dysphagia. Additional file 2: Video 2. Drinking difficulties. Additional file 3:
**Detailed clinical and diagnostic features of the Hungarian Vizslas with idiopathic inflammatory polymyopathy.**

## Abbreviations

CIGMR: Centre for integrated genomic medical research
CK: Creatine kinase
CoI: Inbreeding coefficient
DLA: Dog leukocyte antigen
EMG: Electromyography
IBD: Inflammatory bowel disease
MG: Myasthenia gravis
MHC: Major histocompatibility complex
MMM: Masticatory muscle myositis
MRI: Magnetic resonance image
STIR: Short time inversion recovery
VIP: Vizsla idiopathic inflammatory polymyopathy
