# Supplementary material for: Clinical features of idiopathic inflammatory polymyopathy in the Hungarian Vizsla
Source: BMC Vet Res. 2015 Apr 21;11:97. doi: 10.1186/s12917-015-0408-7 (PMC4414416; doi:10.1186/s12917-015-0408-7)
Supplement: Additional file 3: — Detailed clinical and diagnostic features of the Hungarian Vizslas with idiopathic inflammatory polymyopathy. [file 12917_2015_408_MOESM3_ESM.doc]

Additional file 3 Detailed clinical and diagnostic features of the Hungarian Vizslas with idiopathic inflammatory polymyopathy.

| *Cases* | *Dysphagia**(pharyngeal phase of deglutition)* | *Drinking and eating difficulties**(Oral phase of deglutition)* | *Sialorrhoea* | *Masticatory muscle atrophy* | *Regurgitation* | *Toxoplasma gondii**(serology)* | *Neospora caninum**(serology)* | *Abnormal CK**(>190 IU/L)* | *CK>1000* | *2MAb* | *AchRAb* | *EMG* | *Mega-**oesophagus* | *Aspiration**pneumonia* | *Megaoesophagus diagnosis* | | |
| --- | --- | --- | --- | --- | --- | --- | --- | --- | --- | --- | --- | --- | --- | --- | --- | --- | --- |
|  |  |  |  |  |  |  |  |  |  |  |  |  |  |  | *X-ray* | *Barium* | *Fluoro-**scopy* |
| *1* | *1* | *1* | *1* | *1* | *1* |  |  |  |  |  |  |  |  |  |  |  |  |
| *2* | *1* | *1* | *1* | *1* | *1* |  |  | *1* | *0* |  |  |  | *0* |  | *0* |  |  |
| *3* | *1* | *1* | *1* | *1* | *1* |  |  | *1* | *0* |  |  |  | *0* |  | *0* |  |  |
| *4* | *1* | *1* | *1* | *1* | *1* |  |  |  | *0* |  |  |  |  |  |  |  |  |
| *5* | *1* | *1* | *1* | *1* | *1* |  |  | *1* | *1* |  | *N/A* |  | *1* | *1* | *1* |  |  |
| *6* | *1* | *0* | *0* | *1* | *1* |  |  |  |  |  |  |  |  |  |  |  |  |
| *7* | *1* | *1* | *1* | *1* | *1* | *N* | *N* |  |  | *N* | *N* | *1* |  |  |  |  |  |
| *8* |  | *1* | *1* |  | *1* |  |  |  |  |  |  |  | *1* |  | *1* |  |  |
| *9* | *1* | *1* | *1* | *1* | *1* |  |  | *1* | *1* | *N* |  | *0* | *1* |  | *1* |  |  |
| *10* | *1* | *1* | *1* | *1* | *1* | *N* | *N* | *1* | *1* | *N* | *N* | *1* | *1* |  | *1* |  | *0* |
| *11* | *0* | *0* | *0* | *1* | *0* | *N* | *N* | *0* | *0* | *N/A* |  | *1* | *0* |  | *0* |  |  |
| *12* | *1* | *1* | *1* | *1* | *1* | *N* | *N* | *1* | *1* |  |  |  | *0* |  | *0* |  |  |
| *13* | *1* | *1* | *1* | *1* | *1* |  |  | *1* | *1* |  |  |  | *1* | *1* | *1* |  |  |
| *14* | *1* | *1* | *1* | *0* | *1* | *N* | *N* | *1* | *1* | *N* | *N* |  | *1* |  | *1* |  |  |
| *15* | *1* | *1* | *1* | *1* | *1* |  |  | *0* | *0* | *N/A* | *N* | *1* | *1* | *1* | *1* |  |  |
| *16* | *1* | *1* | *1* | *1* | *0* |  |  |  |  | *N* |  |  |  |  |  |  |  |
| *17* | *1* | *1* | *1* | *1* | *1* |  |  | *1* | *0* | *N* | *N* |  | *0* |  | *0* |  | *0* |
| *18* | *1* | *1* | *1* | *1* | *1* |  |  |  |  |  |  |  | *1* |  | *1* | *1* |  |
| *19* | *0* | *0* | *0* | *1* | *0* |  |  | *1* | *0* |  |  |  |  |  |  |  |  |
| *20* | *1* | *1* | *0* | *1* | *0* |  |  |  |  |  |  |  |  |  |  |  |  |
| *21* | *1* | *1* | *0* |  | *1* |  |  |  |  |  |  |  |  |  |  |  |  |
| *22* | *1* | *1* | *1* | *0* | *1* | *N* | *N* | *1* | *0* | *N* | *N* | *1* | *0* |  | *0* |  |  |
| *23* | *1* | *1* | *1* | *1* | *1* |  |  | *1* | *0* | *N* | *N* |  | *0* |  | *0* |  | *0* |
| *24* | *1* | *1* | *1* |  | *1* |  |  | *1* | *1* | *N* | *N* | *1* | *0* |  | *0* |  |  |
| *25* | *1* | *1* | *1* | *1* | *0* |  |  | *0* | *0* | *N* | *N* |  | *0* |  | *0* | *0* |  |
| *26* |  | *1* |  | *1* |  |  |  | *1* | *0* | *N* | *N* |  |  |  |  |  |  |
| *27* | *0* | *1* | *1* | *1* | *0* | *N* | *N* |  |  | *N* | *N* | *0* | *0* |  | *0* |  |  |
| *28* | *1* | *1* | *1* | *0* | *1* |  |  | *1* | *1* |  |  |  | *0* |  | *0* |  |  |
| *29* | *1* | *1* | *1* | *1* | *1* | *N* | *N* | *1* | *1* | *N* | *N* |  | *1* |  | *1* |  |  |
| *30* | *1* | *1* | *1* | *1* | *1* | *N* | *N* |  | *0* |  |  |  | *0* |  | *0* |  |  |
| *31* | *1* | *0* | *1* | *1* | *0* |  |  |  |  |  |  |  |  |  |  |  |  |
| *32* | *1* | *1* | *1* | *1* | *1* | *N* | *N* | *0* | *0* |  |  |  | *1* | *1* | *1* |  |  |
| *33* | *1* | *1* | *1* | *1* | *1* |  |  |  |  | *N* | *N* |  |  |  |  |  |  |
| *34* | *1* | *1* | *1* | *1* | *1* |  |  |  |  |  |  |  |  |  |  |  |  |
| *35* | *1* | *0* | *1* | *0* | *1* |  |  | *1* | *1* | *N* | *N* |  |  |  |  |  |  |
| *36* | *1* | *1* | *1* | *1* | *0* |  |  |  |  |  |  |  | *0* |  | *0* |  |  |
| *37* | *0* | *0* | *1* | *1* | *1* |  |  | *1* | *1* |  | *N* | *1* | *1* |  | *1* |  |  |
| *38* | *1* | *1* | *1* | *1* | *1* |  |  | *1* | *1* | *N* | *N* |  | *1* |  | *1* |  |  |
| *39* |  | *1* | *1* |  |  |  |  |  |  |  |  |  |  |  |  |  |  |
| *40* |  |  |  | *1* |  | *N* | *N* | *1* | *1* | *N/A* | *N* |  |  |  |  |  |  |
| *41* | *1* | *1* | *1* |  | *1* |  |  | *1* | *1* | *N* | *N* | *1* | *1* |  | *1* |  | *1* |
| *42* | *1* | *1* | *1* | *1* | *1* |  |  |  |  |  |  |  |  |  |  |  |  |
| *43* | *1* | *1* | *1* | *1* | *1* |  |  |  |  |  |  |  | *0* | *1* | *0* |  |  |
| *44* | *1* | *1* | *1* | *1* | *1* | *N* | *N* | *1* | *0* | *N* | *N* | *1* | *1* |  | *1* |  |  |
| *45* | *1* | *1* | *1* | *1* | *1* | *N* | *N* | *1* | *1* |  | *N* | *1* | *0* |  | *0* | *0* |  |
| *46* | *1* | *1* | *1* | *1* | *1* | *N* | *N* | *1* | *0* | *N* |  | *1* |  |  |  |  |  |
| *47* | *1* | *1* | *1* | *1* | *1* | *N* | *N* | *1* | *0* | *N* | *N* | *1* | *0* | *1* | *0* |  |  |
| *48* | *1* | *1* | *1* | *1* | *1* | *N* | *N* |  |  | *N* | *N* |  | *0* |  | *0* |  |  |
| *49* | *1* | *0* | *0* | *1* | *0* |  |  | *1* | *1* |  |  |  |  |  |  |  |  |
| *50* | *1* | *1* | *1* | *1* | *0* |  | *N* | *1* | *0* | *N* |  | *1* |  |  |  |  |  |
| *51* | *1* | *1* | *1* | *1* | *1* | *N* | *N* | *1* | *1* | *N* | *N* | *1* |  |  |  |  |  |
| *52* | *1* | *1* | *1* | *1* | *1* | *N* | *N* |  |  | *N* | *N* |  | *0* | *1* | *0* |  | *0* |
| *53* | *1* | *1* | *1* | *1* | *1* |  |  | *1* | *1* |  | *N* |  | *1* |  | *1* | *1* |  |
| *54* | *1* | *1* | *1* | *1* | *1* | *N* | *N* | *1* | *1* | *N* | *N* | *1* | *1* |  | *1* |  | *1* |
| *55* | *1* | *1* | *1* | *1* | *1* |  |  | *1* | *1* |  |  |  | *0* |  | *0* | *0* |  |
| *56* | *1* | *1* | *1* |  | *1* |  |  |  |  |  | *N* |  | *1* | *1* | *1* |  |  |
| *57* | *1* | *1* | *1* | *1* | *1* | *N* | *N* | *1* | *0* | *N* | *N* | *1* | *1* | *1* | *1* | *1* | *1* |
| *58* | *1* | *1* | *1* | *1* | *1* |  |  | *1* | *1* |  |  | *1* | *1* | *1* | *1* | *1* | *1* |
| *59* | *1* | *1* | *1* | *1* | *1* |  |  |  |  |  |  |  | *0* | *1* | *0* |  |  |
| *60* | *1* | *1* | *1* | *1* | *1* |  |  | *1* | *0* |  |  | *1* | *1* |  | *0* |  | *1* |
| *12* | *1* | *1* |  | *1* | *1* |  |  | *1* | *0* |  | *N* |  | *0* |  | *0* | *0* |  |
| *62* | *1* | *1* | *1* |  | *1* | *N* | *N* | *1* | *1* |  |  | *1* | *1* |  | *0* |  | *1* |
| *63* | *1* | *1* | *1* | *1* | *1* |  |  | *0* | *0* |  |  |  | *1* |  | *1* |  |  |
| *64* | *1* | *1* | *1* | *1* | *1* |  |  | *1* | *0* | *N* | *N* |  | *1* | *1* | *1* |  |  |
| *65* | *1* | *1* | *1* | *1* | *1* |  |  |  |  |  |  |  | *1* | *1* | *1* |  |  |
| *66* | *1* | *1* | *1* | *1* |  |  |  |  |  |  | *N* |  | *1* |  | *1* |  |  |
| *67* | *1* | *1* | *1* | *1* |  |  |  |  |  |  |  | *1* | *0* |  | *0* | *0* | *1* |
| *68* | *1* | *1* | *1* | *1* |  |  |  |  |  |  |  |  | *0* |  | *0* |  |  |
| *69* | *1* | *1* | *1* | *1* | *1* | *N* | *N* | *1* | *0* |  |  |  | *1* | *1* | *1* |  |  |
| *70* | *1* | *1* | *1* | *1* | *1* | *N* | *N* | *1* | *1* | *N* |  | *0* | *0* |  | *0* |  |  |
| *71* | *1* | *1* | *1* | *1* | *1* | *N* |  | *0* | *0* | *N/A* |  |  |  |  |  |  |  |
| *72* | *1* | *1* | *1* | *1* | *1* |  |  |  |  |  |  |  |  | *1* |  |  |  |
| *73* | *1* | *1* | *1* | *1* | *1* |  |  |  |  |  |  |  |  |  |  |  |  |
| *74* | *1* | *1* |  |  | *1* |  |  |  |  |  |  |  | *1* | *1* | *1* |  | *0* |
| *74* | *1* | *1* | *1* | *1* | *1* |  |  |  |  |  |  |  |  |  |  |  |  |
| *77* | *1* | *1* | *1* | *1* | *1* |  |  | *1* | *1* | *N* | *N* |  |  |  | *1* | *1* |  |
| *77* | *1* |  | *1* | *1* | *1* |  |  |  |  |  | *P* |  |  |  |  |  |  |
| *78* | *1* | *1* | *1* | *1* | *1* | *N* | *N* | *1* | *1* |  |  |  | *1* | *1* |  |  |  |
|  | *70* | *69* | *68* | *66* | *62* | *25* | *25* | *47* | *25* | *33* | *34* | *20/23* | *28/52* | *17* | *26* | *10* | *12* |

1= **present ;** 0= **absent or normal;** empty cell= **results not available or test not performed**. 2MAb= **2M antibody test,** AchRAb= **Acetylcholine receptor antibody test,** N= **normal,** P= **positive,** N/A**= test performed and result not available.**
